# Diarrheal Disease in Rural Mozambique: Burden, Risk Factors and Etiology of Diarrheal Disease among Children Aged 0–59 Months Seeking Care at Health Facilities

**DOI:** 10.1371/journal.pone.0119824

**Published:** 2015-05-14

**Authors:** Tacilta Nhampossa, Inacio Mandomando, Sozinho Acacio, Llorenç Quintó, Delfino Vubil, Joaquin Ruiz, Delino Nhalungo, Charfudin Sacoor, Arnaldo Nhabanga, Ariel Nhacolo, Pedro Aide, Sónia Machevo, Betuel Sigaúque, Abel Nhama, Karen Kotloff, Tamer Farag, Dilruba Nasrin, Quique Bassat, Eusebio Macete, Myron M. Levine, Pedro Alonso

**Affiliations:** 1 Centro de Investigação em Saúde de Manhiça (CISM), Maputo, Mozambique; 2 Instituto Nacional de Saúde, Ministério de Saúde, Maputo, Mozambique; 3 Faculdade de Medicina, Universidade Eduardo Mondlane, Maputo, Mozambique; 4 Barcelona Center for International Health Research (CRESIB, Hospital Clínic-Universitat de Barcelona), Barcelona, Spain; 5 Center for Vaccine Development (CVD), University of Maryland School of Medicine, Baltimore, Maryland, United States of America; Aga Khan University Hospital Nairobi, KENYA

## Abstract

**Background:**

Diarrheal disease remains a leading cause of illness and death, particularly in low-income countries. Its burden, microbiological causes and risk factors were examined in children aged 0–59 months living in Manhiça, rural southern Mozambique.

**Methods:**

Trends of diarrhea-related burden of disease were estimated during the period 2001–2012. A prospective, age-stratified and matched (by age, gender and geographical origin), case-control study was conducted during 2007–2011. Clinical, epidemiology, anthropometric measurement and fecal samples obtained from recruited children were used to estimate moderate-to-severe diarrhea (MSD) weighted attributable fractions.

**Results:**

Over the last decade the incidence of acute diarrhea has dropped by about 80%. Incidence of MSD per 100 child years at risk for the period 2007–2011 was 9.85, 7.73 and 2.10 for children aged 0–11, 12–23 and 24–59 months respectively. By adjusted population attributable fractions, most cases of MSD were due to rotavirus, *Cryptosporidium*, ETEC ST (ST only or ST/LT), *Shigella* and Adenovirus 40/41. Washing hands and having facilities to dispose child’s stools were associated with a reduced risk of MSD, while giving stored water to the child was associated with an increased risk of MSD.

**Conclusions:**

Despite the predominantly decreasing trends observed throughout the last decade, diarrheal diseases remain today a major cause of morbidity among children aged 0–59 months living in this rural Mozambican area. Rotavirus, *cryptosporidium*, *Shigella*, ETEC ST and Adenovirus 40/41 were the most important aetiologies of MSD. Thus, well-known preventive strategies such as washing hands, improving the treatment of stored water, having facilities to dispose children stools, and accelerating the introduction of the rotavirus vaccine should be promoted on a wider scale to reduce the current burden of diarrheal diseases.

## Introduction

Diarrheal disease remains a major contributor to illness and death among children less than five years in developing countries. Indeed, pediatric diarrheal disease still accounts for over 800.000 annual deaths globally, *circa* 11% of the 7.6 million estimated annual global child deaths [1]. However, a review of studies from the past two decades suggests that mortality from diarrhea has been steadily decreasing worldwide, mainly due to the implementation of effective control programs and an improved socioeconomic situation [1–4].

Diarrhea may be caused by infectious organisms, including viruses, bacteria, protozoa, and helminths [5]. Nonetheless, the epidemiological, etiological agents and risk factors of diarrhea vary greatly depending on country, region and community, so their knowledge is essential to inform prevention and control programs. Information from Mozambique is relatively scarce, but confirms that diarrheal diseases are a significant contributor to morbidity and mortality, especially among younger children [6–9]. In addition, to our knowledge no study has specifically investigated moderate-to-severe diarrhea (MSD) episodes. This is the first study that simultaneously investigated environmental factors, primary caretaker’s characteristics and the microbiological etiology of MSD in Manhiça, a rural area of Southern Mozambique. In this country, approximately 65% of the population lives in rural settings [10] and are at similar risk of having diarrhea. We hereby present an analysis of the burden, microbiologic etiology and risk factors associated to MSD among all patients <5 years of age included as part of the Global Enteric Multicenter Study (GEMS), a larger, matched, case-control study on the etiology and epidemiology of diarrheal diseases conducted between the years 2007–2011 in Manhiça district [11].

## Methods

### Study area and population

The study was conducted, in the District of Manhiça, a rural area located 80 kilometers north of the capital of Mozambique, Maputo. The climate is subtropical with two distinct seasons: a warm and rainy season from November to April and a generally cooler and drier season during the rest of the year. The average annual temperature ranges from 22°C to 24°C and annual rainfall ranges from 600 to 1000mm. Community prevalence of HIV/AIDS in Manhiça is amongst the highest in the world, with prevalence rates in women in child-bearing age as high as 40% in the district [12]. Diarrhea is the third leading cause of hospital admission among children aged 0–14 years and the fourth leading cause of death among children between 12 and 59 months [6], according to verbal autopsies performed in the area. The Manhiça district has about 150,000 inhabitants, and the *Centro de Investigação em Saúde da Manhiça* (CISM) runs a demographic surveillance system (DSS) in this district since 1996, involving intensive and regular monitoring of a population of about 92,000 inhabitants in an area of around 500km^2^. About a fifth (19%) of the study area inhabitants are children aged <5 years [13]. A round-the-clock morbidity surveillance system, covering both pediatric outpatient and hospital admission was established in 1996 at the Manhiça District Hospital-MDH (the main facility and the only one with admission facilities) and has progressively integrated five other rural health posts [14]. Clinical data for all children under 15 years of age are routinely collected by a trained medical officer or physician using standardized forms.

### Study design

This manuscript describes the results of two different studies. In the first study we estimated the incidence of acute diarrhea episodes (see definition below) in children aged 0–59 months admitted to MDH between the years 2001 and 2012 based on an ongoing health facility morbidity surveillance system related to the DSS. And secondly, a case-control study was conducted between December 2007, and October 2011. All children aged 0–59 months belonging to the DSS population who sought care at the health facilities within DSS area were screened for diarrhea, defined as three or more loose stools within the previous 24h. Study clinicians assessed each child with diarrhea for eligibility. To be included, the episode had to be acute (onset after ≥7 diarrhea-free days), acute (onset within the previous 7 days), and fulfil at least one of the following criteria for moderate-to-severe diarrhea: sunken eyes (confirmed by parent or caretaker as more than normal); loss of skin turgor (abdominal skin pinch with slow [≤2 s] or very slow [>2 s] recoil); intravenous hydration administered or prescribed; dysentery (visible blood in loose stools); or admission to hospital with diarrhea or dysentery [15]. For each child with MSD, one to three healthy control children (no story of diarrhea in the previous 7 days) were randomly selected from the neighborhood in which the case resided using the DSS database within 14 days of presentation of the diarrhea respective case. Controls were also matched by age and gender. The following data were recorded at the time of the query: demographic, socioeconomic status, breastfeeding patterns (exclusive, partial or no breastfeeding), water and sanitation environmental, clinical presentation and anthropometric measurement (weight, height and Mid Upper Arm Circumference-MUAC-), in addition to history of taking antibiotics in the preceding 4 hours before the interview was recorded. We conducted a follow-up visit about 60 days after enrolment to assess the child’s vital status, capture interim medical events, and repeat anthropometric measurements. Case fecal samples were collected within 12 hours of registration of the diarrheal episode, and control samples within 14 days after case enrolment. Once collected, samples were kept in a cool box until processed. Children were not enrolled if unable to produce fecal samples within the established first 12 hours or prior to the initiation of antibiotics. Each fecal specimen comprised a whole stool specimen (in screw top fecal specimen cups carried in Styrofoam boxes with cold packs), a fecal swab in Modified Cary Blair medium in a plastic screw top test tube, and a fecal swab in buffered glycerol saline in a screw top test tube [16]. Additionally, if antibiotics were to be given to patients before stool was produced, we obtained two rectal swabs for bacterial culture pending passage of the whole stool for the remaining assays.

### Specimen processing for pathogen detection

Fecal specimens were platted on media for detection of bacterial pathogens according to standard methods [17]. Bacterial agents (*Salmonella*, *Shigella*, *Campylobacter*, *Aeromonas*, and *Vibrio* spp) were detected using conventional culture techniques. Three putative *Escherichia coli* colonies from every stool were pooled (from 2-day growth on MacConkey plates, several lactose-fermenting bacterial colonies resembling E. coli were picked and tested using motility indole ornithine medium) and analysed by multiplex PCR that detect targets for enterotoxigenic (ETEC), entero aggregative (EAEC), enteropathogenic (EPEC), and entero haemorrhagic *E coli* (EHEC). The following gene targets defined each *E coli* pathotype: ETEC (either *eltB* for heat-labile toxin [LT], *estA* for heat-stable toxin [ST], or both), ST-ETEC (either *eltB* and *estA*, or *estA* only), typical EPEC (*bfpA* with or without *eae*), atypical EPEC (*eae* without either *bfpA*, *stx1*, or *stx2*), EAEC (*aatA*, *aaiC*, or both), and EHEC (*eae* with *stx1*, *stx2*, or both, and without *bfpA*). Commercial immunoassays detected rotavirus (ELISA ProSpecT Rotavirus kit, Oxoid, Basingstoke, UK) and adenovirus (ProSpecT Adenovirus Microplate (Oxoid); adenovirus-positive samples were tested for enteric adenovirus serotypes 40 and 41 (Premier Adenoclone kit, Meridian Bioscience, Cincinnati, OH, USA). Norovirus (genogroups I and II), sapovirus, and astrovirus were detected using multiplex reverse transcriptase (RT) PCR [17]. Individual commercial immunoassays (TechLab, Inc, Blacksburg, VA, USA) detected *Giardia lamblia*, *E*. *histolytica/E*. *dispar*. and *Crytosporidium* spp.

### Definitions

Primary caretaker education was stratified in two groups: no formal education (no education or did not complete primary education) or some formal education (at least completed primary education). In order to assess socio-economic status, wealth quintiles were generated following the methods described by Filmer et al[18]. Access to improved water was defined according to the household’s use of the following types of water supply for drinking: piped water, public tap, borehole or pump, protected well, protected spring or rainwater. Improved water sources did not include vendor-provided waters, bottled water, tanker trucks or unprotected wells and springs, rivers or ponds. Treating water behavior was assessed according to a predefined number of possible answers in the questionnaire, which included: “leave water in the sun to disinfect, filter through cloths, ceramic or other filter, chlorine use, boiling. Stored water was considered when water was placed in a container (clay pot, cooking pot, jerrycan, plastic bottle or oil drum) for any duration after it was collected from the source. Water availability was assessed as: all the time, several hours every day, a few times per day and less frequent than a few times per week. Improved sanitation facilities for the household included: connection to a public sewer or septic system, pour-flush latrine, simple pit latrine, or ventilated improved pit latrine. Unimproved sanitation facilities included public or shared latrine, open pit latrine (traditional pit latrine), or bucket latrine. Improved facilities for handling stools included burying faeces or disposing faeces in toilet or latrine. Hand washing assessed by closed questions whose answer alternatives were yes or no.

### Statistical analysis

**Fig 1:**
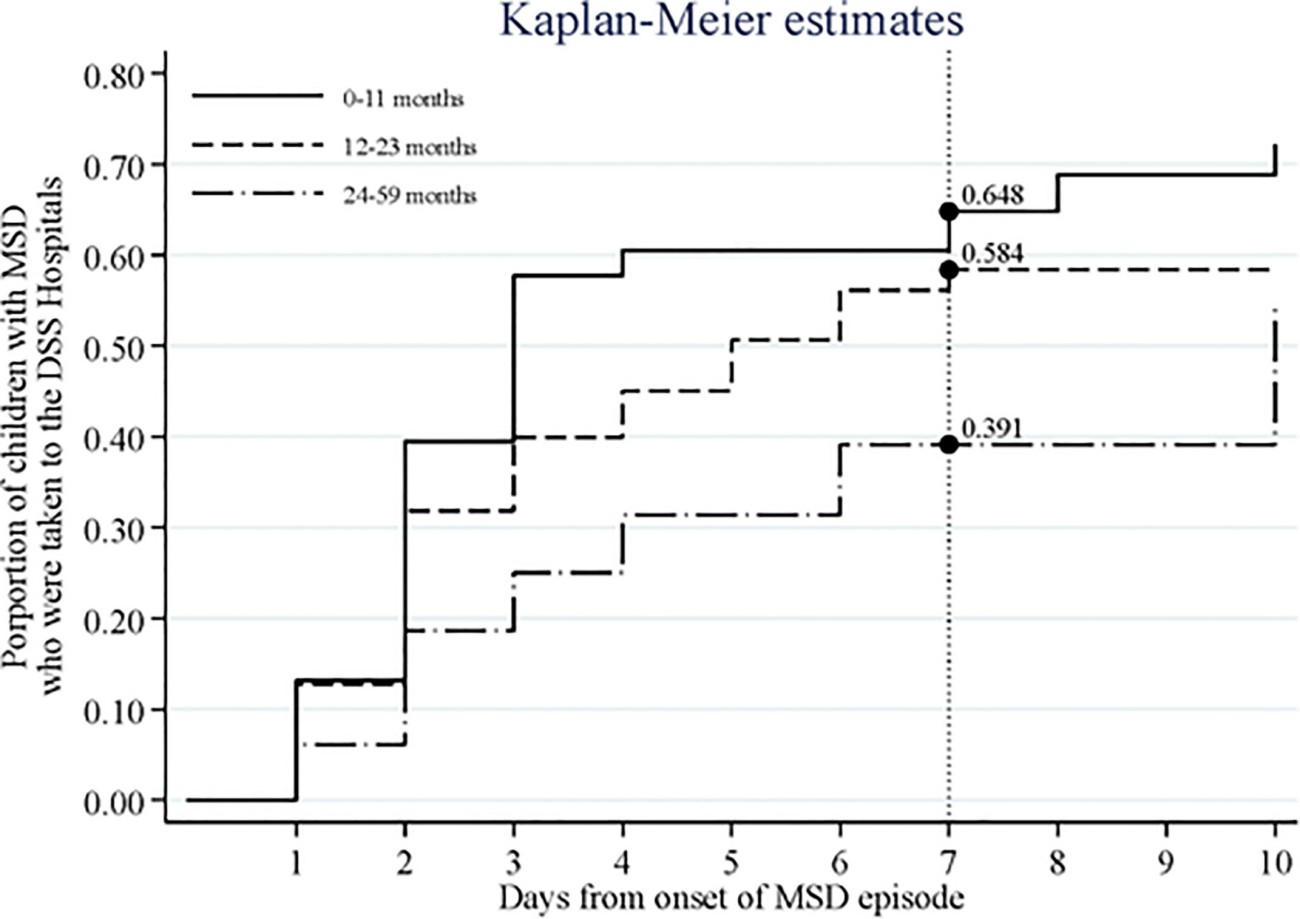
Proportions of visits to DSS hospitals within 7 days of onset of moderate-to-severe diarrhea (*r* value): results from serial surveys about health utilization and attitudes during 2009–2011.

Analyses were conducted using STATA software (version 12.0) (StataCorp LP, College Station, TX, USA). The study targeted three age strata: infants (0–11 months), toddlers (12–23 months), and children (24–59 months); for each site and age stratum, we used the median population from DSS rounds during the case-control study for analyses. To estimate the Minimum community-based incidence rates (MCBIR), deriving from passive case detection of diarrhea episodes being detected at the hospital, the time at risk was calculated as the number of person years at risk since the beginning of the time at risk until the end of follow-up. The beginning of time at risk was defined for each child as the first day of study period (January 1, 2001) or date of birth, whatever occurred later. The end of follow-up was defined for each child as the last day of study period (December 31, 2012) or the date of death or the date of becoming 5 years, whatever occurred earlier. Children did not contribute to the numerator/denominator for a period of 7 days after each episode of hospital-detected diarrhea (a new episode of diarrhea meant that ≥7 days had elapsed since the last occurrence of diarrhea) or when they were outside the study area. Negative binomial regression models were estimated to compare incidence rates between age-groups or calendar years. For the case-control analysis, variables with missing values in their matched pair were not included and differences between matched pairs were evaluated by paired t-test or Sign test of matched pairs for continuous variables and by Exact McNemar significance probability test or Symmetry (asymptotic) test for categorical variables. Associations with MSD were estimated by conditional logistic regression, and multivariable models were estimated using a step-down process including all those variables with a percentage of missing or unmatched values less than 5% and with p-value < 0.2 in the crude analysis and adjusted for socio-demographic and nutritional variables, other pathogens and interactions between pathogens. Weighted attributable fractions (AF) to estimate pathogen-specific disease burden (expressed as number of cases and incidence rate) of MSD (unadjusted/ adjusted), annual attributable cases and attributable incidences were calculated for all variables with a positive association with MSD. The weighted attributable fraction provides an indication of what the percentage reduction in the incidence rate of a disease could be in a given population if the exposure were eliminated altogether. According to the study protocol, cases of MSD were included in approximately equal numbers each fortnight, regardless of the number of cases having visited health facilities within the DSS area. This was taken into account and weighted attributable fractions were also estimated, using weights defined as the inverse of the sampling fraction (number of eligible cases divided by the number of enrolled cases in each fortnight) [15]. These weights were calculated separately for cases with and without dysentery, to avoid any bias from overrepresentation or underrepresentation of cases with dysentery. We combined data for two or more adjacent fortnights to have at least one case with dysentery and at least one case without dysentery in each time period. Using the methods developed by Bruzzi et al, [19] we estimated the population attributable fractions (unadjusted/adjusted and weighted/unweighted) for all other pathogens included in the model, in order to determine the fraction of MSD to be avoided, if the pathogens associated with MSD could be perfectly controlled. In contrast to the odds ratio, this measure takes into account the pathogen’s prevalence. To estimate the burden of MSD, repeated surveys were conducted during the case-control study to find out the proportion of cases that usually goes to the health facilities within one week of onset of MSD (called r). We combined the data from these surveys and we weighted them by sampling weights, based on the number of children in each age-sex stratum according to information from the DSS during each round [15]. Then, we estimated the values of r (Fig 1) and its variances for each age stratum by Kaplan Meier analysis. The annual number of cases of MSD in the population was calculated as the average number of eligible cases per year divided by r. The annual cases divided by the median of the population gave the MSD incidence rate. Such incidence rates, derived from the case-control study will be called throughout the manuscript as Health Care Utilization and Attitudes survey (HUAS)-inferred incidence rates, so as to differentiate them from those calculated through passive case detection of diarrhea at the hospital, which will be termed MCBIR. To calculate the number of cases and the incidence rates attributable to a specific pathogen, the total cases and incidence rates were multiplied by the pathogen's weighted and adjusted AF.

### Ethical statement

This study is part of the Global Enteric Multicenter Study (GEMS), a large multicenter study conducted in six other developing countries investigating the etiology and epidemiology of diarrheal disease in infants and young children. The overall protocol and informed consent were both approved by the National Bioethics Committee of Mozambique (CNBS), the ethics committee of the Hospital Clinic of Barcelona and the Institutional Review Board at the University of Maryland. After informing the objectives and characteristics of the study a written informed consent was obtained from the child’s caretaker. One copy of the consent form was left with the caretaker and the other retained in locked cabinets at CISM.

## Results

### Historical trends and burden of diarrheal disease

**Fig 2:**
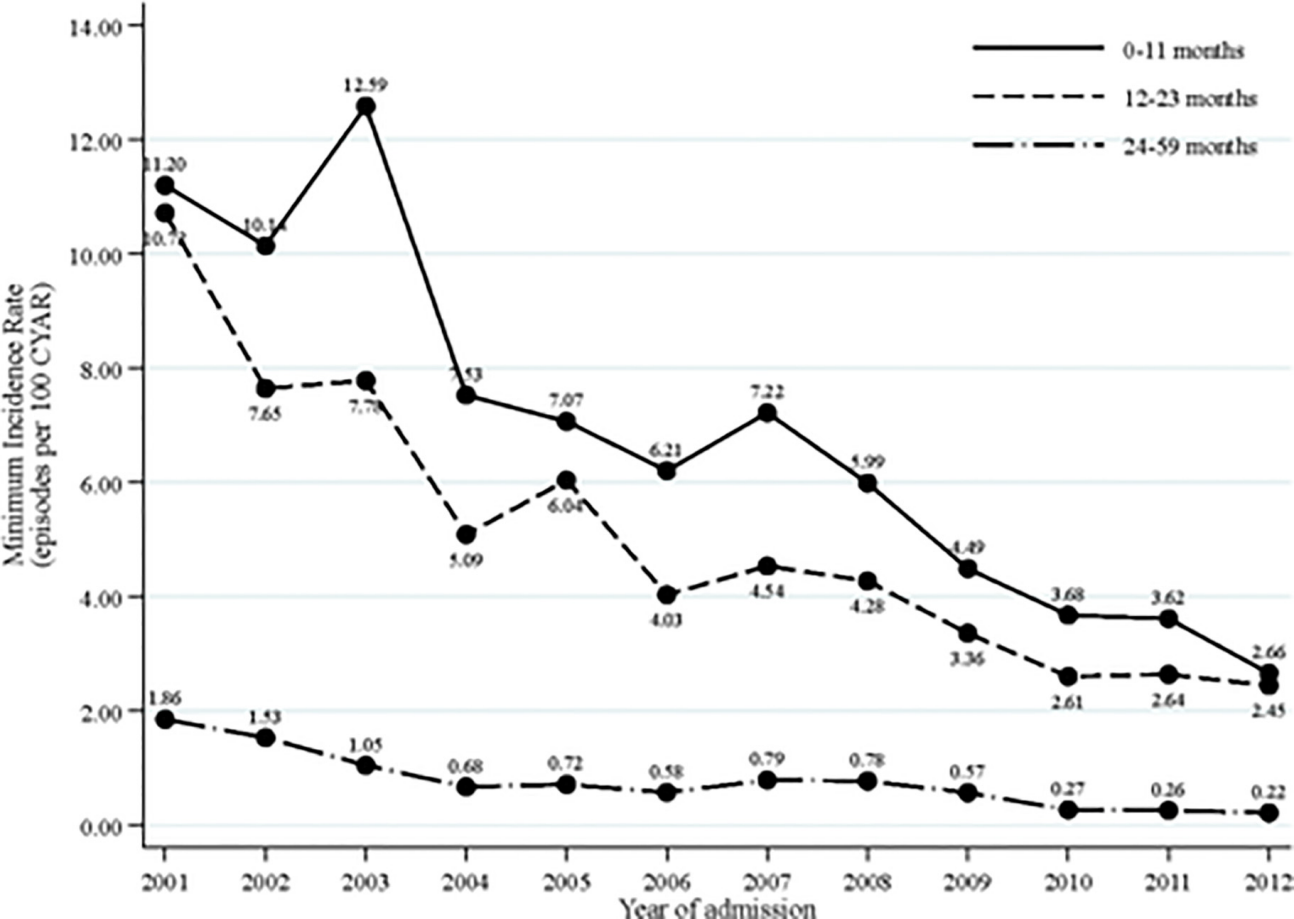
Minimum community-based incidence rates trends of hospitalized acute diarrhea episodes according to age during 2001–2012.

Yearly minimum community-based incidence rates (MCBIR) of acute diarrheal hospital admissions during 2001–2012 are shown in Fig 2. Throughout the decade MCBIR have been highest in the 0–11 month’s age group and lowest in the 24–59 months. All age groups have shown a steady decline that represents an 88% drop over the twelve years period in the older age group, a 77% in the 12–23 months group and a 76% in the youngest group. The risk of acute diarrhea decreased with increasing age (12–23 vs. 0–11 months, IRR = 0.72, 95%CI: 0.67–0.77; 24–59 vs. 0–11 months, IRR = 0.10, 95%CI: 0.10–0.11; p<0.001). Point estimates of weighted annual rates for MSD during 2007–2011 (HUAS-inferred incidence rates) delivered from the surveillance and the case-control study were 9.85 episodes in infants (0–11 months), 7.73 in children aged 12–23 months and 2.10 per 100 CYAR in children aged 24–59 months.

### The case control study

**Fig 3:**
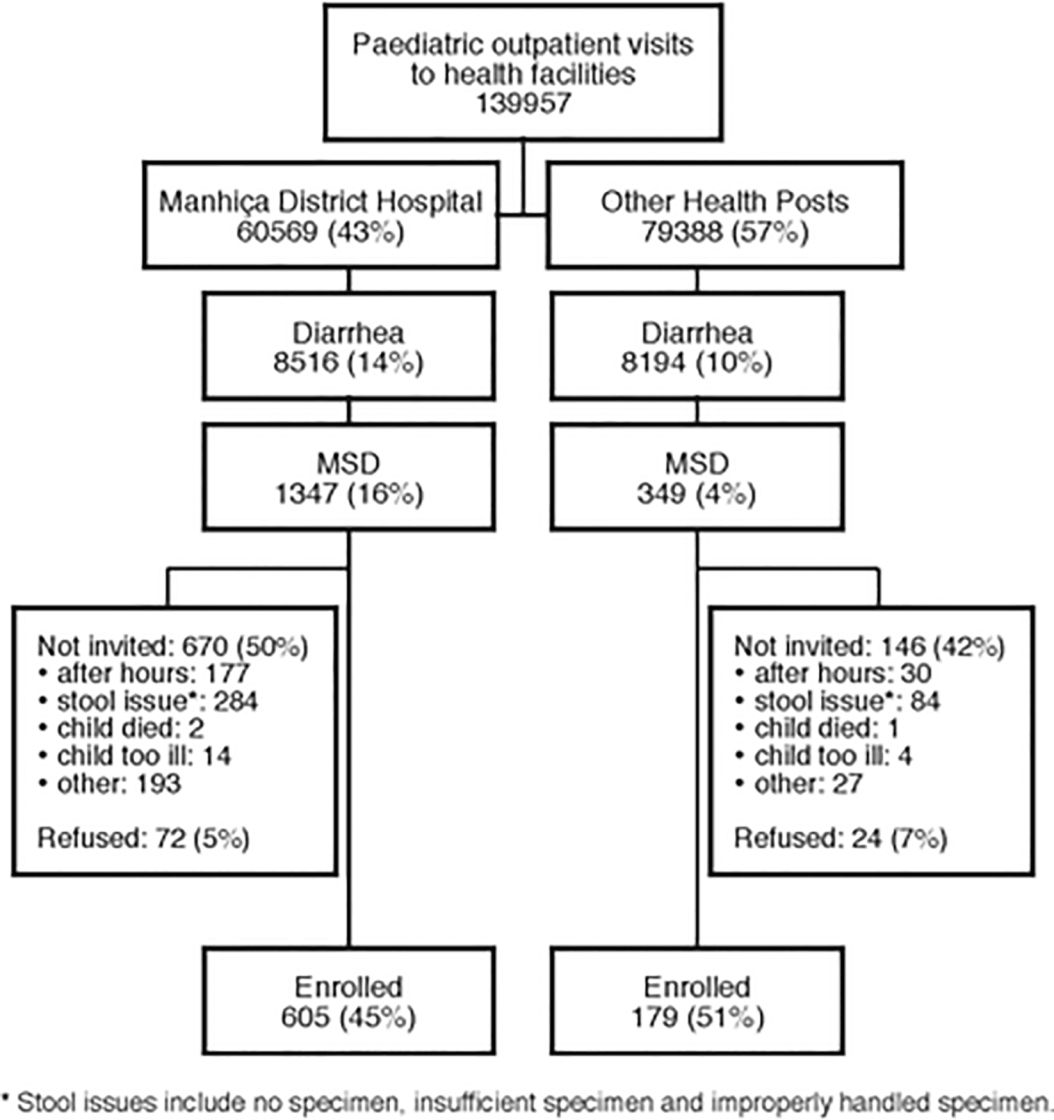
Study profile showing number of patients and reason for not recruting (December 2007–November 2011).

Fig 3 presents the study profile. During the 4-years study period (December 2007-October 2011), a total of 1696 children aged <5 years presented with MSD criteria and among these, 48% (816/1696) were not invited to participate due to the following reasons: no stool within 12 hours of registration (21–26%), stool issues (42–57%), child dying (0.3–0.6%), child too ill (2–2.7%) and other reasons (18–29%). Six percent (96/1696) of the eligible children refused to participate. We finally recruited 784 children with MSD and 1545 matched children with no diarrhea. As per the criterion used for defining a MSD case, enrolled children were more likely to have been hospitalized with diarrhea or dysentery (65%) followed by increased sunken eyes (51%), wrinkled skin (36%) and dysentery (15%).

#### Demographic and socioeconomic characteristics

The characteristics of the enrolled children in the study are shown in Table 1. Because of the matched selection procedure, MSD cases were nearly identical to control children with respect to sex and age. The caretaker respondent was the mother in 93% of cases and in 90% of the controls. More than two thirds (76% vs. 73%) of the primary caretakers were on the group of lower educational level. The median number of people per household was 6. When we compared socio-economic indicators in cases and controls, controls in the three age groups had a higher mean wealth quintile, albeit differences were not statistically significant. Having animals in the compound, less frequently reported in cases compared to their controls, was only associated with MSD in children aged 12–23 months (p<0.001).

**Table 1:** Baseline characteristics of cases and controls included in the case control study.

Variables	0–11 months			12–23 months			24–59 months		
	Casesn (%)	Controlsn (%)	P value	Casesn (%)	Controlsn (%)	P value	Casesn (%)	Controlsn (%)	P value
	N = 431	N = 861		N = 233	N = 502		N = 120	N = 232	
Age in months: median (IQR)	7 (5–9)	7 (5–9)		16 (14–19)	16 (13–19)		31 (27–37)	30 (27–37)	
Child sex (male)	259 (60)	517 (60)		132 (57)	302 (60)		69 (57)	149 (64)	
Child primary caretaker (mother)	416 (97)	854 (99)	<0.001	214 (92)	485 (97)	0.007	103 (86)	203 (88)	0.922
Caretaker formal education									
No formal education	316 (74)	606 (71)		183 (79)	371 (75)		92 (77)	177 (77)	
Some formal education	112 (26)	251 (29)	0.632	48 (21)	123 (25)	0.280	27 (23)	54 (23)	0.922
Family size: median (IQR)	6 (4–8)	7 (5–9)	0.003	6 (5–8)	6 (5–9)	0.836	6 (5–8)	7 (5–9)	0.029
Mean wealth quintile (SD) [n]	1.90 (1.42) [426]	2.07 (1.39) [855]	0.140	1.87 (1.41) [233]	1.93 (1.42) [502]	1.000	2.03 (1.54) [119	2.19 (1.45)[229]	0.766
Animals in compound	359 (83)	758 (88)	0.126	191 (82)	453 (90)	0.012	99 (82)	195 (84)	0.820
Water availability (not always per day)	230 (53)	272 (32)	<0.001	130 (56)	176 (35)	<0.001	59 (49)	82 (35)	0.007
Access to improved water	360 (84)	715 (83)	0.401	194 (83)	410 (82)	0.345	99 (82)	193 (83)	0.682
Give stored water to child	394 (91)	671 (78)	<0.001	227 (97)	471 (94)	0.061	113 (93)	216 (93)	0.324
Treating water habit	51 (12)	57 (7)	0.002	11 (5)	25 (5)	0.557	7 (6)	9 (4)	0.777
Facility to dispose child´s stool	208 (49)	511 (60)	<0.001	187 (81)	472 (95)	<0.001	115 (96)	228 (99)	0.066
Improved facility for household stool	38 (9)	54 (6)	0.056	14 (6)	21 (4)	0.212	13 (11)	18 (8)	0.433
Washing of hands habit									
Before eating	406 (94)	817 (95)	0.737	204 (88)	482 (96)	<0.001	111 (92)	219 (94)	0.254
Before cooking	265 (61)	731 (85)	<0.001	135 (58)	449 (89)	<0.001	71 (59)	193 (83)	<0.001
Before preparing baby’s food	113 (26)	599 (70)	<0.001	71 (30)	368 (73)	<0.001	31 (26)	147 (63)	<0.001
After defecating	370 (86)	767 (89)	0.159	204 (88)	456 (91)	0.257	106 (88)	210 (91)	0.636
After handling animals	12 (3)	257 (30)	<0.001	4 (2)	146 (29)	<0.001	5 (4)	60 (26)	1
After cleaning child feces	107 (25)	495 (57)	<0.001	51 (22)	261 (52)	<0.001	23 (19)	10 (44)	<0.001
Breastfeeding									
No	12 (3)	15 (2)		90 (39)	143 (28)		98 (82)	225 (97)	
Partially	246 (57)	495 (57)		103 (44)	265 (53)		12 (10)	7 (3)	
Exclusively	173 (40)	351 (41)	0.227	39 (17)	94 (19)	<0.001	10 (8)	0	0.011
Height-for-age z-score: mean (SD)	-1.21 (1.42)	-1.09 (1.22)	0.043	1.75 (1.42)	-1.55 (1.18)	0.048	-1.55 (1.33)	-1.68 (1.21)	0.756

IQR = Interquartile range. SD = Standard deviation. *Significant difference, by conditional logistic regression (p≤0.05).

#### Water exposure

The main sources of water to the study population were public tap (36% for cases vs. 31% for controls) and borehole (25% for cases vs. 24% for controls). The vast majority of the households (83% for cases vs. 83% for controls) had access to improved water, but this did not seem associated with a lower risk of MSD in any of the different age-groups (Table 1). Most of the population, (90% of the caretakers) reported fetching water daily with a median of 5 (Interquartile range IQR = 4–7) and 6 (IQR = 4–8) trips per day for cases and controls respectively. From this, 12% of the cases and 22% of the controls needed over 30 minutes to get to the water source. Only a small proportion (6–9%) of the caretakers reported that they used to treat drinking water and among these, the use of chlorine (55% for cases vs. 75% for controls) and boiling water (45% for cases vs. 25% for controls) were the most commonly used methods. Treating water habits were associated with an increased risk of MSD in children aged 0–11 months (OR 1.93; 95%IC 1.27–2.92, p = 0.002). Giving stored water to the child was also associated with an increased risk of MSD in children aged 0–11 months (p<0.001).

#### Hygiene practices

Hand washing appears as a strong protective factor against MSD (Table 1). With regards to disposal of human waste, only 2% of the households did not have any facility whatsoever and did not report sharing or using facilities from other households. Most households used traditional pit toilet, (about 90% of cases and 92% of controls) rather than improved facilities such as improved latrines. Somehow counter intuitively, the use of improved facilities for handling household stools was not associated with reduced risk of MSD in the univariate analysis. However, the use of facilities to dispose child’s stools was associated with a reduced risk of MSD in children aged less than two (p<0.001).

#### Breastfeeding and nutritional characteristics

Breastfeeding pattern was not shown to protectively affect MSD among pediatric patients by univariate analysis. Moreover, breastfeeding was shown to be a quasi-universal practice, with 97% of children in both groups practicing it (Table 1). The mean height-for-age z-score among both cases and controls was considerably below the WHO reference for all age groups and, with one exception, deviated further from the reference at 12–23 months age group. However, the effect on the risk of MSD was small and of borderline significance (p = 0.042 and p = 0.48 for children aged 0–11 months and 12–23 months, respectively).

#### Risk factors by multivariate analysis

Independent risk factors for MSD according to age group by multivariate analysis are shown in Table 2. Significantly, giving stored water to the child was independently associated with an increased risk of MSD particularly for infants (p<0.001) and young children (p = 0.049). On the other hand, washing of hands habit, mainly after handling animals or before preparing baby’s food were found to be protective factors for MSD in all age groups. Having facilities to dispose child’s stool was associated with a lower risk of the occurrence of MSD.

**Table 2:** Independent risk factors for moderate-to-severe diarrhea by multivariate analysis.

Variables	OR	95%CI	*P*
0–11 months			
Child primary caretakers (mother)	0.07	0.02–0.29	<0.001
Number of sleeping rooms	0.95	0.90–0.99	0.014
Give stored water to child	6.92	3.57–13.39	<0.001
Treating water habit	2.19	1.19–4.04	0.012
Improved facility for household stool	2.70	1.45–5.26	<0.001
hand washing before cooking	0.35	0.24–0.52	<0.001
Hand washing before prepare baby’s food	0.25	0.16–0.37	<0.001
Hand washing after handling animals	0.08	0.03–0.19	<0.001
Hand washing after cleaning child feces	0.57	0.38–0.85	0.006
12–23 months			
Child primary caretakers (mother)	0.27	0.08–0.9	0.033
Water availability (not always per day)	1.88	1.10–3.21	0.021
Give stored water to child	3.88	1.01–14.93	0.049
Facility to dispose child’s stool	0.35	0.16–0.76	0.008
Hand washing hands before eating	0.39	0.16–0.94	0.035
Hand washing hands before cooking	0.12	0.06–0.24	<0.001
Hand washing hands before prepare baby’s food	0.33	0.19–0.58	<0.001
Hand washing after handling animals	0.03	0.00–0.24	<0.001
Breastfeeding			
No	1		
Partially	0.43	0.23–0.83	
Exclusively	1.68	0.82–3.44	0.032
24–59 months			
Facility to dispose child’s stool	0.03	0.00–0.31	0.004
Hand washing hands before cooking	0.43	0.21–0.86	0.016
Hand washing hands before prepare baby’s food	0.23	0.11–0.47	<0.001
Hand washing after cleaning child feces	0.28	0.12–0.65	0.003

#### Microbiological studies

We identified at least one enteropathogen in 666 (85%) children with MSD and in 1214 (76%) of the controls; and two or more agents in 376 (48%) cases and in 596 (37%) controls (p<0.001). Table 3 summarizes the frequency of pathogens detected in fecal samples in both the MSD and control groups. The etiologic agents detected more frequently includedrotavirus, *G lamblia*, *Cryptosporidium*, EAEC aatA, and *E*. *histolytica/E*. *dispar*, although there were differences across age groups. Crude and adjusted analyses of pathogens associated to MSD according to age group and consequent attributable fractions are shown in Table 4.

**Table 3:** Frequency of pathogens in stool samples of children with moderate-to-severe diarrhea and the control group.

Pathogens	0–11 months			12–23 months			24–59 months		
	Cases	Controls	P	Cases	Controls	P	Cases	Controls	P
	N = 431	N = 861		N = 233	N = 502		N = 120	N = 232	
	n (%)	n (%)		n (%)	n (%)		n (%)	n (%)	
Protozoa									
*G*. *lamblia*	41 (10)	152 (18)	<0.001	64 (28)	228 (46)	<0.001	42 (35)	115 (50)	0.020
*Cryptosporidium*	84 (20)	86 (10)	<0.001	44 (19)	46 (9)	<0.001	11 (9)	18 (8)	0.229
*E*. *histolytica/E*. *dispar*	39 (9)	79 (9)	0.607	26 (11)	52 (10)	0.837	15 (12)	28 (12)	0.920
Viruses									
Rotavirus	182 (42)	139 (16)	<0.001	52 (22)	91 (18)	0.014	12 (10)	27 (12)	0.821
Adenovirus 40/41	9 (2)	8 (1)	0.087	6 (3)	5 (1)	0.150	-	-	-
Adenovirus non 40/41	4 (1)	22 (3)	0.134	4 (2)	7 (1)	0.389	0 (0)	6 (3)	-
Norovirus	19 (4)	38 (4)	0.578	10 (4)	25 (5)	0.286	4 (3)	12 (5)	0.109
Sapovirus	7 (2)	24 (3)	1.124	3 (1)	11 (2)	0.360	0 (0)	3 (1)	-
Astrovirus	7 (2)	11 (1)	0.600	6 (3)	10 (2)	0.477	1 (1)	4 (2)	0.477
Bacteria									
ETEC ST (ST only or ST/LT)	20 (5)	19 (2)	0.040	29 (12)	16 (3)	<0.001	8 (7)	12 (5)	0.612
ETEC LT	12 (3)	58 (7)	0.005	17 (7)	32 (6)	0.409	3 (2)	7 (3)	0.818
EAEC aatA	95 (22)	184 (21)	0.563	27 (12)	29 (6)	0.032	9 (8)	14 (6)	0.906
EAEC aaiC	32 (7)	42 (5)	0.249	18 (8)	37 (7)	0.889	11 (9)	12 (5)	0.261
EAEC aaiC/aatA	23 (5)	47 (5)	0.548	15 (6)	26 (5)	0.693	1 (1)	9 (4)	0.121
EPEC typical	43 (10)	67 (8)	0.277	17 (7)	35 (7)	1.000	5 (4)	13 (6)	0.441
EPEC atypical	6 (1)	19 (2)	0.345	1 (0)	11 (2)	0.170	2 (2)	4 (2)	1.000
*Shigella*	6 (1)	1 (0)	1.000	18 (8)	2(0)	<0.001	20 (17)	0 (0)	-
*Salmonella non-Typhi*	6 (1)	6 (1)	0.115	2 (1)	0 (0)	1.000	-	-	-
*Vibrio cholerae 01*	-	-	-	4 (2)	1 (0)	0.145	9 (8)	0 (0)	1.000
*Aeromonas*	5 (1)	0.065	0.065	1 (0)	0 (0)	1.000	0 (0)	1 (0)	1.000
*Campylobacter*	24 (6)	0.339	0.339	9 (4)	14 (3)	0.289	0 (0)	2 (1)	-

P value not estimated due to no observation in the case group

**Table 4:** Crude and multivariate analysis, weighted attributable fractions and incidence of pathogens significantly associated with moderate-to-severe diarrhea.

	UnadjustedOR (95%CI) AF (95%CI)		AdjustedˡOR (95%CI) AF (95%CI)		Incidence rate per 100CYAR (95%CI)
0–11 months					
MSD total	-	-	-	-	9.85 (8.41–11.30)
MSD-attributable	-	-	-	46.03 (41.79–50.26)	4.54 (3.75–5.32)
Rotavirus	5.35 (3.87–7.38)	33.27 (30.80–35.74)	6.00 (3.65–9.87)	34.75 (31.30–38.20)	3.42 (2.82–4.03)
*Cryptosporidium*	2.62 (1.83–3.74)	12.78 (9.96–15.60)	3.67 (2.06–6.54)	15.26 (11.96–18.56)	1.56 (1.15–1.97)
12–23 months					
MSD total	-	-	-	-	7.73 (6.32–9.15)
MSD-attributable	-	-	-	35.62 (29.14–42.10)	2.75 (2.04–3.47)
*Shigella*	20.82 (4.78–90.70)	8.42 (7.79–9.04)	19.79 (3.33–117.67)	8.65 (7.83–9.57)	0.67 (0.53–0.81)
ETEC ST (ST only or ST/LT)	4.63 (2.37–9.06)	9.91 (8.08–11.74)	33.50 (5.80–193.54)	3.68 (-15.37–22.73) ^2^	0.28 (-1.19–1.76) ^2^
Adenovirus 40/41	2.46 (0.72–8.39)	1.69 (0.27–3.12)	14.05 (2.06–95.84)	2.73 (2.33–3.13)	0.21 (0.16–0.26)
Rotavirus	1.68 (1.11–2.55)	-	3.30 (1.60–6.79)	-	-
24–59 months					
MSD total	-	-	-	-	2.10 (1.45–2.76)

(1): Adjusted for socio-demographic and nutritional variables, other pathogens and pathogen’s interaction

(2): Negative values at lower limit are due to a negative interaction between *ETEC ST* and *Rotavirus* in the multivariate model

AF: attributable fraction; CYAR: child years at risk; OR: odd ration; CI: Confidence Interval

Importantly, 53.97–64.38% of all MSD could not be attributable to any of the pathogens investigated. Rotavirus and *Cryptosporidium* were significantly associated with MSD in infants, while ETEC ST (ST only or ST/LT), *Shigella*, adenovirus 40/41 and rotavirus were associated to MSD in children aged 12–23 months. Our models could not confirm the association of any specific pathogen with MSD in the older age group. There was a negative interaction between Rotavirus and ETEC ST (ST only or ST/LT): OR = 0.01 (95% CI: 0.00–0.11) in children aged 12–23 months and paradoxically, *G lamblia* was consistently associated with a lower risk of MSD in all age groups.

## Discussion

In this study we have documented the sharp decline throughout a period of over a decade in the incidence of diarrhea in Mozambican children. Nevertheless, despite the predominant decreasing trend, diarrheal disease remains a major cause of morbidity in children aged less than five in Manhiça district as in most of the country. Thus, our findings reinforce the need to improve the implementation of general sanitation practices (such as washing hands and having facilities to dispose child’s stools safely), particularly among children aged 0–23 months who presented the highest risk. With the identification of the pathogens independently associated with MSD and their respective pathogen-specific AF, we estimated that 35.62–46.03% of MSD could be reduced with specific interventions against those particular pathogens such as effective vaccines. This implies however that more than half (53.97–64.38%) of all MSD could not be attributable to any of the pathogens investigated. Incidences in the case-control study (HUAS-inferred incidence rates) appear higher than calculated MCBIRs, due to the fact that MCBIR correspond to diarrhea cases seeking care at the hospital, while MSD incidences were calculated using all eligible MSD adjusted by r [20] (thus total MSD regardless of health facility use).

In the absence of specific interventions, we tend to intuitively associate this predominantly decreasing diarrheal disease trend, with the general improvement in living conditions since the end of civil strife in the early 90῾s. Indeed, Mozambique has experienced in recent years a steady economic growth rate, leading to the reduction of some of its poverty pockets. Such an improvement has likely been translated to better access to safe water and improved sanitation, as the high coverage rates among study participants seem to confirm. Besides this, the also improved access to basic health care, coupled with the practically universal practice of breastfeeding may have also played a major role in the decreasing trends in diarrheal-related morbidity and mortality. However, in this study we have failed to associate economic status and MSD risk. Conditional logistic regression was performed, even though, overmatching may still have affected the precision of odds ratios estimates since case and controls were likely to share the same environmental conditions [21]. Furthermore, we also did not manage to find associations between the formal education level of the primary caretaker and the risk of MSD. This is in contrast with the literature that suggests that the primary caretaker's increasing level of education has been considered an indicator of knowledge or behavior in relation to child health, and thus a protective factor against MSD [22, 23].

The study observed that hygiene practices related with washing hands and having facilities to dispose child’s stool were associated with a reduced risk of MSD. These results are consistent with the literature [11] and reinforce the need to continue promoting the implementation of these two measures to reverse the intolerable toll that diarrhea poses in rural Mozambican settings. Indeed, in this rural setting, traditional pit toilets were the most commonly used facilities to dispose human fecal waste. Only 2% of al households did not have any toilet facility, and this was not found to be a risk factor for MSD. One would imagine that these households could actually practice open defecation or might probably share facilities with neighbouring households. Toilet sharing creates unsanitary and unkempt conditions, which provide conducive environments for vectors and pathogens associated with diarrhea, increasing also the possibility of inter-household transmission[24]. Contrarily, we did find a clear association between having facilities for disposing children stools and a decreased risk of MDS. Thus, the fact that children living in households using improved facilities were at increased risk of developing MSD may reflect an incorrect use of those facilities or insufficient hygienic measures at the household level. It is in any case clear that measures to encourage the proper use of latrines linked to hygiene behavior are probably critical to further decrease the burden of MSD in the area.

Our results also revealed that regularly treating drinking water behavior or access to improved water source (frequently reported) were not protectively associated with the occurrence of MSD. Additionally, having and using stored water appeared to be a major risk factor for MSD, as other authors have suggested [25]. These results are striking and may suggest that, either treating drinking water is incorrectly performed, or that conservation methods employed are not safe (in fact giving stored water to a child increased the odds of MSD). The inherent difficulties in eliminating certain pathogens from water sources (such as for example *Cryptosporidium* or *Giardia* which may persist in water despite the use of conventional water treatment measures), may however help to understand some of these findings[26, 27]. It is also likely that asking directly about “do you usually treat drinking water at home?” led to a significant response bias that was possibly conditioned by the fact that the caretakers wanted to impress a good behavior related to water quality. While the confirmation of the different primary caretaker answers was done by interviewer’s observation of sanitation facilities or water source during community controls enrollment, confirmation and record of chlorine result test was only performed in the follow-up visit and verification of the boiling water process was almost impossible. Therefore, our findings reinforce the need to improve the implementation of diarrhea control measures with emphasis on monitoring of water quality and adjusting the levels of chlorination in water supply.

In sharp contrast to studies that have consistently shown that stunting is a significant risk factor for childhood diarrheal disease [28], we failed to demonstrate this association in the present study. However, although stunting was evaluated as a potential risk factor, it is important to note that it could also be the result of previous diarrhea episodes. This involves the measurement of cause and effect at the same time, introducing the problem of temporal ambiguity in establishing causal relationships. Mean height-for-age z-scores among both cases and controls were considerably below the WHO reference for all age groups and, with one exception, deviated further from the reference at 12–23 months age group.

Breast-feeding, specially if this is the only source of nutrition, has been shown to protect children against diarrhea in Africa as elsewhere in the developing world [29]. Surprisingly, when controlled by other confounders, the particular breastfeeding pattern was not shown to protectively affect MSD among pediatric patients. Although the rate of breastfeeding is high and weaning practices were not evaluated, low maternal education probably does not allow adequate dietary practices. In developing countries, diarrhea usually presents its peak incidence in the second year of life, overlapping with inadequate weaning practices to the introduction of external food, and interacting with an increase exposure of the toddler to contaminated food and to lack of sanitation and personal and domestic hygiene.

In this study we confirmed rotavirus as the main cause of diarrhea in children aged less than two in Manhiça district. Rotavirus accounted for more than a third of MSD cases in infants, and its incidence rates markedly exceeded those of others pathogens. Despite the abovementioned decreasing trends, diarrhea caused by rotavirus remains frequent and imposes a substantial burden on the health system, so a vaccine against rotavirus could hasten the decline of diarrheal disease morbidity. The high incidence found in this study is in close accordance with that of various regions of Sub-Saharan Africa, where rotavirus has been described as the leading cause of diarrheal disease [30]. Contrariwise, in a study conducted in Manhiça more than 10 years ago [7], rotavirus was only detected in 1% of symptomatic children younger than 5 years of age, and this was probably due to the low sensitivity and specificity of the used assays. In the present study, adenovirus 40/41 was comparatively rare; nevertheless it does not necessarily exclude this pathogen as an important cause of diarrhea in children, particularly because adenovirus 40/41 remained significantly associated to MSD in children aged 12–23 months.

*Cryptosporidium* was found to be the second most important cause of diarrhea in our series. Globally, *Cryptosporidium*, is recognized as an important cause of diarrhea, both in immunocompetent individuals[31, 32], and also among immunocompromised ones [27, 33]. In the developing world, traditional detection methods have a low sensitivity, and the increased utilization of quantitative PCR and antigen detection tools is uncovering the real burden of this pathogen. In the developed world, some of the sources of transmission include the consumption of contaminated water and food[26], or a close contact with recreational contaminated waters[27]. In our setting, close contact with potentially contaminated water or food may have been an important source of infections. Thus, without a Cryptosporidium vaccine, and facing considerable diagnostic challenges and ineffective treatment in young infants, minimizing the overall environmental burden (contaminated water/food) needs prioritization. Furthermore, the high underlying community burden of HIV infection[12], another well characterized risk factor for cryptosporidiosis [34] may have played also an important role.

*Shigella*, which is known to cause outbreaks of diarrhea in various communities [35, 36], was just detected in 44 (6%) patients with MSD. Its low detection rate in children without diarrhea 3 (0%), confirms the high probability that, whenever present, this pathogen causes disease. *Shigella flexneri* was the most prevalent serotype identified in our study (80%) and the most frequently detected in previous studies in Manhiça district [37].

The occurrence of *Escherichia coli* strains ranging from 9–30% of the children, is higher than those firstly published in this district in the same age group [7]. However, ETEC ST (ST only or ST/LT) was the only Escherichia coli strain causing MSD in children. The absence of EHEC strains is not surprising and neither is the lower frequency of *Salmonella* and *Campylobacter* found in our study. These data are consistent with those from other neighboring areas in which these microorganisms were detected in less than 3% of children aged <5 years with diarrhea [7, 38, 39].

*Vibrio cholerae 01* was detected mainly in children aged above two with diarrhea. This age related pattern of pathogens is consistent with reports from studies conducted in other developing countries [40] and should be taken into account when considering appropriate management of childhood diarrhea in Mozambique. As no outbreak of cholera was reported in the area during the time of study, this finding supports the described changing epidemiology tendency of *V*. *cholerae* in recent years in several regions of Mozambique, from the “epidemic disease” to “endemic disease with epidemic peaks” as a result of the cumulative number of asymptomatic carriers at the end of each peak. However, some of the risk factors for MSD found in this analysis, and particularly those related with the treatment and storage of water, are also favorable for the spread of *V*. *cholerae* infections. *V*. *cholerae* was detected in one child without diarrhea demonstrating the occurrence, albeit rare, of asymptomatic carriers in the region. WHO has recommended the use of antibiotic treatment in order to decrease the duration of disease and mortality, and control its transmission. However, and according to Mozambique’s Ministry of Health “the use of antibiotics for cholera treatment is expressly prohibited to avoid emergence of bacterial resistance to antimicrobials” [41]. This rule is carefully checked respected for asymptomatic infection, but clinicians administer antibiotics in case of serious illness.

Two findings concerning the appreciation of the role of the detected pathogens as etiologic agents are noteworthy. First, there were high rates of positive stool samples with potential enteropathogens in children without MSD, a finding that has previously been described for various pathogens, including cryptosporidium [31]. And second, *G lamblia*, also as previously demonstrated [42], was consistently associated with a lower risk of MSD in all age groups. The characteristics of the pathogens (pathogenicity, duration of excretion and interaction with other pathogens), host and environmental factors are recognized to be the justifications for the above results [43], thus future studies should explore in greater depth the abovementioned factors when studying diarrheal etiology, to better understand pathogen causality. Indeed, results from metanalysis have shown that in developing-country settings, the initial or first few, *G lamblia* infections may result in diarrhea but immunity is rapidly acquired, thereupon conferring protection against symptomatic disease when subsequently exposed [44, 45].

While some of the strengths and limitations of the study have been discussed previously by Kotloff et al [15, 46], it seems pertinent to mention other methodological observations arising from the analysis of the strengths and weaknesses of this study that should be taken into account. First, almost half of children with MSD criteria were finally not invited to participate in the study, mainly on account of impossibility of obtaining stool samples. This factor might have contributed to lower estimations of the proportion of MSD attributable to specific pathogens. The possibility however of introducing a major bias should have been minimized by the design of the study, by which the inclusion in the study was conducted using a fortnightly quota, in order to avoid possible effects of seasonality or peaks of infection, recognizing however that not being able to adequately assess the effect of seasonality in pathogen detection is also a limitation of such a design. It is true, however, that the cases which were not recruited on account of developing severe disease or even dying, or due to any other causes, may have introduced a certain recruitment bias to the study. Second, although caretakers were asked about the history of taking antibiotics in the four hours preceding sample collection, it is likely that among a population in which over two thirds of the respondents were in the group of lower educational level, reporting bias for this information may have occurred. Antibiotics not restricted to the 4 hours prior to sampling, and taken in the preceding days may also have affected the performance of detection methods. This may partly explain why 53.97–64.38% of the MDS cases could not be attributable to any of the pathogens investigated. Third, due to the negative effect of the interaction between Rotavirus and ETEC ST (ST only or ST/LT) in children aged 12–23 months, AF of rotavirus become negative despite it was associated to an increased odd of MSD. Finally, mild-diarrhea cases (an early stage of MSD) were not investigated, and could contribute to provide adequate and timely preventive interventions. Irrespective of these limitations, the study constitutes a comprehensive survey of the impact of diarrheal disease and pathogens causing MSD among children aged 0–59 months that gives an overall adequate picture of the current situation of diarrheal disease in a typical rural Mozambican area.

In conclusion, despite the predominant decreasing trend, diarrheal diseases remain a major cause of morbidity mainly among children aged less than two in rural Mozambique. Rotavirus, *cryptosporidium*, *Shigella*, ETEC ST and Adenovirus 40/41 were the most important causes of MSD. Thus, well-known preventive strategies such as washing hands, improving the treatment of water and its adequate storage, and having facilities to dispose child’s stool and accelerating the introduction of rotavirus vaccine should be promoted on a wider scale to reduce the current diarrheal diseases burden. High rates of positive stool samples with potential enteropathogens in children without diarrhea were frequently observed, underlining the difficulties of determining the cause of an episode of MSD. Thus, the role of the detected pathogens as etiologic agents, the acquisition of immunity to some of those pathogens, and the potential impact of concomitant HIV/AIDS infection need to be further investigated among this population.
